# Copepod Foraging on the Basis of Food Nutritional Quality: Can Copepods Really Choose?

**DOI:** 10.1371/journal.pone.0084742

**Published:** 2013-12-26

**Authors:** Stamatina Isari, Meritxell Antό, Enric Saiz

**Affiliations:** Departament de Biologia Marina i Oceanografia, Institut de Ciències del Mar – Consejo Superior de Investigaciones Científicas, Barcelona, Catalunya, Spain; University of Hamburg, Germany

## Abstract

Copepods have been considered capable of selective feeding based on several factors (i.e., prey size, toxicity, and motility). However, their selective feeding behaviour as a function of food quality remains poorly understood, despite the potential impact of such a process on copepod fitness and trophodynamics. In this study, we aimed to evaluate the ability of copepods to feed selectively according to the nutritional value of the prey. We investigated the feeding performance of the calanoid copepod *Acartia grani* under nutritionally distinct diets of the dinoflagellate *Heterocapsa* sp. (nutrient-replete, N-depleted and P-depleted) using unialgal suspensions and mixtures of prey (nutrient-replete vs. nutrient-depleted). Despite the distinct cell elemental composition among algal treatments (e.g., C:N:P molar ratios) and the clear dietary impact on egg production rates (generally higher number of eggs under a nutrient-replete diet), no impact on copepod feeding rates was observed. All unialgal suspensions were cleared at similar rates, and this pattern was independent of food concentration. When the prey were offered as mixtures, we did not detect selective behaviour in either the N-limitation (nutrient-replete vs. N-depleted *Heterocapsa* cells) or P-limitation (nutrient-replete vs. P-depleted *Heterocapsa* cells) experiments. The lack of selectivity observed in the current study contrasts with previous observations, in which stronger nutritional differences were tested. Under normal natural circumstances, nutritional differences in natural prey assemblages might not be sufficiently strong to trigger a selective response in copepods based on that factor alone. In addition, our results suggest that nutritional quality might depend not only on the growing conditions but also on the inherent taxonomical properties of the prey.

## Introduction

For more than half a century, a considerable body of literature has been dedicated to understanding the foraging strategies of copepods. Numerous experimental works, behavioural observations and extensive discussions have attempted to elucidate certain feeding aspects of these tiny, but extremely important, grazers of the planktonic food web. Our current view is that copepods have developed highly selective feeding behaviour [1], [2], [3]. Based on mechanical and chemical perception, copepods are able to detect, appraise, and further ingest or reject prey items based on certain characteristics [4], [5], [6].

The key role of prey size in determining patterns of copepod feeding selectivity is well documented, e.g., [7], [8]; in particular, large cells can be individually handled and selected, whereas small cells appear to be more passively accumulated [1]. Prey motility is also a well-known factor driving the probability of a prey being ingested, affecting encounter rates and conspicuity to potential predators [9], [10] as well as the capability of the prey to escape from a predator attack [11], [12]. 

Copepods are also able to choose their prey on a coarse quality basis; for instance, they may select against extreme dietary options, e.g., “non-food items” and “harmful food”. An effective response in the two-option choice of “food” vs. “no food” has been witnessed in many laboratory studies. For instance, Poulet and Marsot [13] showed that calanoids may discern between artificial microcapsules that do or do not contain algal extract. Similarly, other works have reported a copepod ability to positively discriminate “algal-flavoured” beads or phytoplankton cells from non-nutritious beads [14], [15], [16], [17], [18], detritus and dead cells [18], [19], [20]. As with the “non-food” signal, copepods may also feed selectively when encountering food with harmful properties, e.g., toxic substances. Although the mechanisms involved in this process are equivocal [21], [22], [23], several studies have reported active discriminatory feeding on toxic algae (mostly dinoflagellates) when a non-toxic choice is offered simultaneously [23], [24], [25]. Nevertheless, toxic algae are not always avoided, and an overall impaired feeding activity, associated with the post-ingestion effects of the toxins consumed, can be observed [26].

The ability of copepods to feed selectively in terms of the chemical composition of prey remains much less understood. This is particularly relevant when subtle differences among prey, probably the most likely magnitude of difference to be encountered, are involved [18]. However, the nutritional selective capability of copepods is difficult to assess, both in the field (when copepods are offered a naturally occurring prey assemblage [27], [28]) and in the laboratory (when copepods are offered mixtures of different algae [29]), as several factors may simultaneously govern the selection of prey. To detect the copepod’s ability to select on the basis of prey biochemical composition, other prey properties that may affect selectivity (e.g., size, motility, and toxicity) should be “subtracted”. In this regard, the effects of prey nutritional quality on copepod feeding have mostly been studied using unialgal diets, where grazers were supplied with distinct biochemical types (e.g., different C:N ratios, essential compounds) of a certain algal species [30], [31], [32]. Such studies have yielded rather contradictory outcomes, and only on a few occasions have higher grazing rates for food of better quality been reported [7], [30], [33]. However, single-food trials cannot be considered authentic selection experiments; the latter would require the simultaneous presence of different quality types. To the best of our knowledge, evaluations of selectivity in mixed food suspensions are very rare for both marine calanoid copepods [33] and their freshwater representatives [34]. The two cited studies reported that copepods exhibit selective feeding behaviour when offered mixed suspensions of two clones of an algal species with different chemical compositions, i.e., selecting cells of relatively higher nitrogen content over others characterised by lower N. Although in field experiments such selectivity processes are difficult to discern, the ability of copepods to select prey according to their nutritional quality would have strong effects on copepod fitness, as food chemical composition affects both the egg production [31], [35] [36], and somatic growth of copepods [32], [37]. However, in our opinion, the ability of copepods to discriminate among prey items on a nutritional basis is not sufficiently investigated, particularly if we take into account the highly diversified prey-pool of copepods and the suggested prey-specific feeding response of copepods when issues of nutritional quality are under question, e.g., [38,39].

Here, we studied the feeding performance of the marine calanoid copepod *Acartia grani* as a function of food quality, focusing on the ability to feed selectively according to the nutritional load of the prey. In particular, we examined the impacts of nitrogen (N) and phosphorus (P) dietary deficiencies on the feeding process, offering cells of a dinoflagellate (*Heterocapsa* sp.) grown under distinct nutrient conditions (nutrient replete, N-limited and P-limited). We produced cell types that, despite the distinct nutrient loads, were virtually indistinguishable in terms of size and morphology, and managed to discriminate them when in mixtures by fluorescent vital staining. Feeding experiments were conducted with both single and mixed suspensions of the distinctly nutrient-loaded prey, at two distinct food levels (moderate and almost saturating). The nutritional quality of the food was evaluated directly through a stoichiometric approach but also indirectly by assessing its impact on copepod egg production.

 Our work adds new knowledge to the field of copepod feeding behaviour as a function of the nutritional quality of the prey, not only due to the conduction of grazing incubations in mixtures of distinct prey types but also because we contemplated the role of dietary P-deficiency, which has been poorly studied in marine copepods [37]. Although phosphorus limitation has traditionally been thought to be rare in marine environments, recent studies indicate that it may be of higher relevance than previously believed [40], [41] [42], and therefore might affect copepod performance considerably. We also discuss the underlying processes governing copepod feeding selection on the basis of the nutritional quality of the prey and the potential implications that such a capability might have for the trophodynamics of marine copepods.

## Materials and Methods

### Ethics statement

No specific permission was required for activities related to field sampling. The field location was not privately owned or protected, and neither endangered nor protected species were involved.

### Culture of algae

We aimed to produce similarly sized cell types of a selected algal taxon with distinct nutritional qualities (C:N:P ratios) that could be used for the copepod feeding experiments. As prey, we chose a strain of the autotrophic marine dinoflagellate *Heterocapsa* sp., isolated from Barcelona Harbour waters in August 1988. Our choice was based on existing information about this strain’s biochemical composition in relation to nutrient conditions [43], [44].

*Heterocapsa* sp. growing exponentially in f/2 medium were inoculated (100 mL; ca. 60000 cells mL^-1^) into 2-L Pyrex flasks containing 1 L of autoclaved 0.2-μm filtered seawater at three nutrient conditions: one replete (regular nutrients of f/2 medium [45]) and the other two depleted by a factor of 20 compared to f/2, either in terms of nitrogen (N/40) or phosphorus (P/40). The seawater used for the cultures had very low nutrient content (N and P concentrations were 0.32% and 0.26% of the f/2 medium, respectively) and did not significantly affect the culture medium conditions. The three batch cultures were maintained in a cold room (18±1°C) under a 12:12 h light:dark cycle (100 μmol m^-2^ s^-1^), and their growth was monitored for 12 days. Cell concentration and biovolume were determined with a Coulter Multisizer III once per day, always at the same time (ca. 14:00 h) to avoid variations due to the light:dark cycle [46]. To establish the most suitable time to harvest the cultures ("harvest day") for setting up the copepod feeding experiment (i.e., before large changes in cell size occur among nutrient treatments), preliminary trials were conducted to determine the temporal variation in cell size and cell concentration in the cultures.

### Stoichiometric composition of algae and dissolved nutrient analysis

The elemental composition (C, N and P) of the algae grown in the different nutrient conditions was assessed during both the preliminary trials and main experiments. Determinations were made on the “harvest day” and the following day (in case staining was applied, see below). Duplicate aliquots (15 mL for C-N analysis, 5 mL for P analysis) of known cell concentration were filtered onto pre-combusted 25 mm diameter GF/F filters (450°C, 6 h). Samples for C and N analysis were dried for 24 h at 60°C and kept in a desiccator until analysis with a CNHS elemental analyser (LECO-932). Filters for particulate phosphorus analysis were frozen at -80°C immediately after filtration and later subjected to orthophosphate acid persulphate oxidation and analysis [47].

The dissolved inorganic nutrient concentration (nitrate, nitrite, phosphate) in the seawater and culture media was analysed with a SEAL Analytical AA3 automatic analyser following the analytical methods described in [47]. 

### Labelling of algae for discrimination in mixed food suspensions

To discriminate algae of virtually identical morphology but different stoichiometric compositions when in mixed suspensions, we used the vital fluorescent stain Cell tracker® Blue CMAC (7-amino-4-chloromethylcoumarin, Molecular Probes Inc.). This fluorochrome permits cell viability, is retained through several generations without being transferred to adjacent cells in a population, and has been used before in copepod feeding experiments [23], [25]. We always incubated the cells of the f/2 treatment at a final stain concentration of 5 μM for 4 h at 18° C [25]. To remove the excess stain, stained cells were centrifuged for 10 min at 1000 rpm, the supernatant was removed, and the cells were re-suspended in filtered seawater and left overnight to recover. The following day, visual observations confirmed the recovery and normal swimming of the algae. Epifluorescence microscopy verified that the cells were stained and fluoresced bright blue when excited by UV light at 354 nm; however, after processing we evidenced an unexpectedly high presence of free thecas of *Heterocapsa* sp. Posterior trials indicated that the loss of thecas was due not to staining but to mechanical stress during centrifugation; different combinations of time and speed centrifugation did not resolve the issue. However, further tests indicated that the staining and the presence of thecas in the food suspension had no influence on copepod feeding activity (Text S1, Figure S1), allowing the use of the procedure.

### Copepod culture

For our work, we collected eggs from the culture of the calanoid copepod *A. grani* kept at the Institut de Ciències del Mar (CSIC) and reared them to adulthood at 18±1°C. Throughout their development, the copepods were fed ad libitum a suspension of *Rhodomonas salina* grown exponentially in f/2 medium. Only female copepods that had matured within the previous week were used in the experiments.

### Single suspension feeding experiments

Single suspension experiments were set up to assess the feeding rates of *A. grani* on *Heterocapsa* sp. cells of different elemental compositions (f/2, N/40, P/40). The three food types were offered at two levels: ca. 500 cells mL^-1^ (165 μg C L^-1^) and ca. 2000 cells mL^-1^ (660 μg C L^-1^); these food concentrations were, respectively, limiting and near saturation according to previous functional response data (Figure S2). Cultures for preparing the respective food suspensions were monitored daily and harvested before large changes in cell size occurred among nutrient treatments ("harvest day", see Results). Prior to the experiments, the copepods were preconditioned for 24 h in suspensions of *Heterocapsa* sp. (growing exponentially in f/2 medium) at the respective cell concentrations.

For each experimental treatment, six 625-mL Pyrex screw-cap bottles were used: batches of females (18 and 32 for the low and high concentrations, respectively) were added to three bottles (3 x Cop), whereas the other three bottles served as initial (1 x Init) and control (2 x Contr) bottles. Initial bottles were immediately sampled (50 mL samples) and preserved in 2% Lugol’s solution, and the rest were incubated on a slowly rotating plankton wheel (0.2 rpm) for 5 h at 18°C. We chose such a short incubation time to hinder any changes in the cell properties that could occur during the incubation time; cell removal at the end of the incubation did not exceed 20%. After the incubation time, the contents of the bottles were carefully poured through a 200-µm mesh, the number and condition of copepods checked (mortality was negligible), and 50-mL samples preserved as above. At least 500 cells were counted in each sample by inverted microscopy. Clearance and ingestion rates as well as the average food concentrations were computed according to Frost [8].

### Feeding selectivity experiments

Two experiments were conducted to determine the feeding selectivity of *A. grani* on algal mixtures with cell types of different stoichiometric composition. In the first one, the effect of N-limitation was examined by providing a mixed suspension of f/2 and N/40 cells (1:1), whereas in the second, P-limitation was investigated by offering a mixed suspension of f/2 and P/40 cells (1:1). Suspensions were prepared from cells collected as above ("harvest day", i.e., same cell size); because the staining procedure (involving centrifugation and overnight recovery) was applied only to the f/2 cells, to avoid bias, the other cell types (N/40 and P/40) were handled similarly but without stain. Each food level consisted of ten 625-mL Pyrex screw-cap bottles (2 x Init, 4 x Contr, 4 x Cop). The number of copepods per bottle, the duration of incubation and other general procedures were as in the single-food experiments; food was offered at two concentrations, respectively ca. 500 and 2000 cells mL^-1^. Initial and final samples were preserved in 2% Lugol’s solution but also in glutaraldehyde (1% final concentration). Glutaraldehyde samples were stored at 4°C in the dark for 2 h, filtered onto 2 μm black polycarbonate filters and finally stored frozen until counting. Under epifluorescence microscopy, 1500 cells per filter were identified on average, covering ca. 6% of the filter area. The proportions of each cell type (f/2 vs. either N/40 or P/40) from epifluorescence microscopy were applied to the Lugol’s counts (total cell numbers) and clearance and ingestion rates computed as described above. Prey preference was assessed by Chesson’s selectivity index α [48]:

$$\alpha =\frac{r_{i}/n_{i}}{\sum_{j=1}^{m}r_{j}/n_{j}}$$

where *r*_*i*_ is the frequency of prey *i* in the diet, *n*_*i*_ is the frequency of prey *i* in the food suspension, and *m* is the number of prey types. This index varies between 0 and 1, with α_*i*_=0.5 indicating non-selective feeding towards prey *i*, α_*i*_ >0.5 indicates positive preference for prey *i*, and α_*i*_ <0.5 indicates discrimination against prey *i*.

### Egg production experiments

We evaluated the impacts of the three different diets (f/2, N/40 and P/40) on the reproductive output of *A. grani*. Adult females were preconditioned in the respective diets at both food levels for four consecutive days. Groups of 20 and 30 females were pipetted into 2300-mL Pyrex screw-cap bottles for the low and high food levels, respectively; twelve males were also added to each bottle to ensure fertilisation. During the preconditioning period, the copepods were daily sieved out and placed in fresh suspensions (discarding dead animals if present), and the spawned eggs (including empty shells) were counted. On the fifth day, five replicated groups of copepods (five females and two males) per treatment were incubated in 625-mL Pyrex screw-cap bottles. After 24 h, the bottles were taken down and the eggs were counted. To ensure similarity in size and the difference in quality among the algal types during the whole preconditioning and experimental periods, five different series of the three algal treatments were set up and harvested with one-day lags for the corresponding incubations. 

## Results

### Algal growth and C:N:P stoichiometry

**Figure 1:**
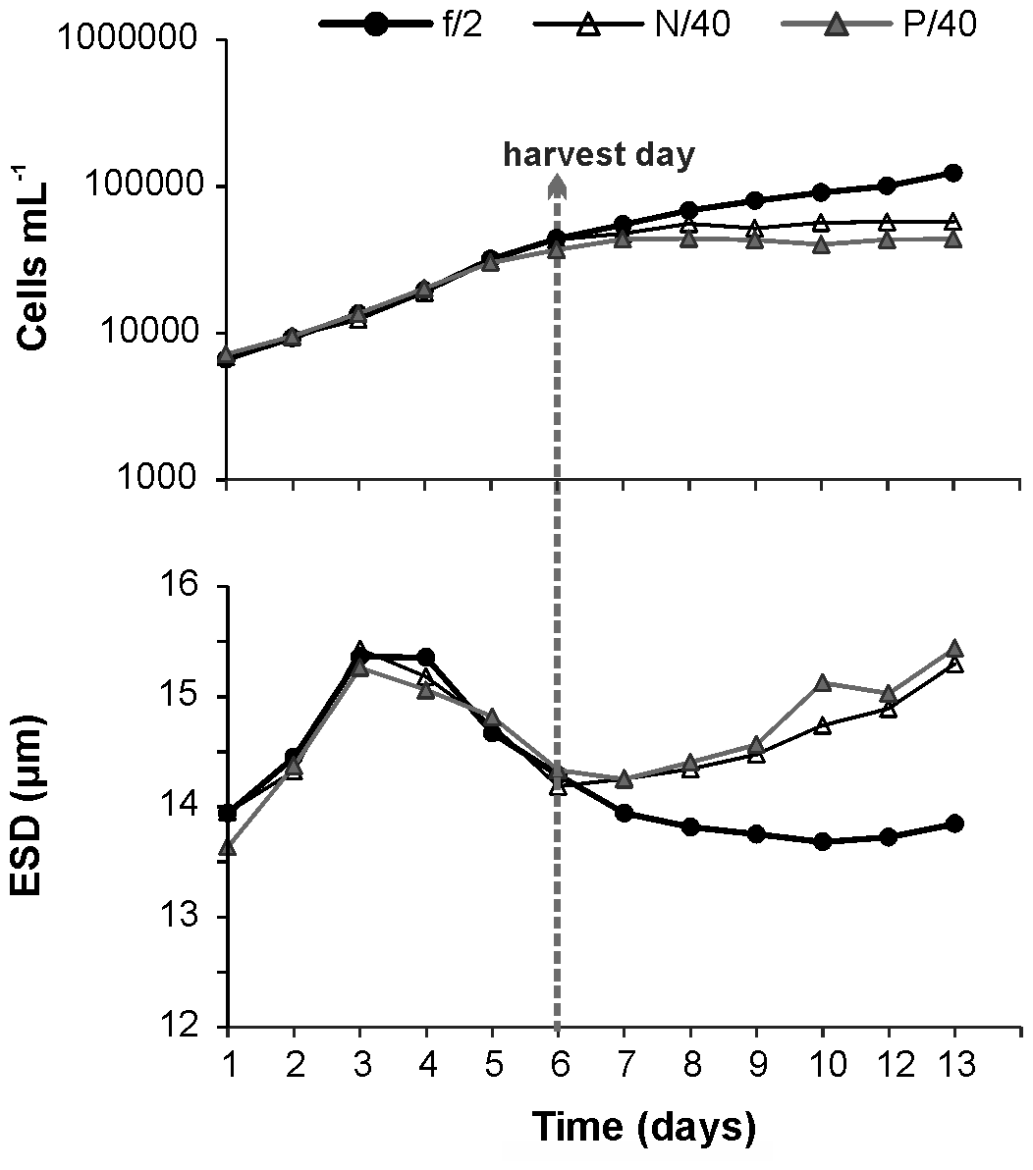
Growth of the *Heterocapsa* sp. cultures. Temporal evolution of cell concentration and size (ESD, equivalent spherical diameter) of the autotrophic dinoflagellate *Heterocapsa* sp. when cultured under three nutrient treatments (f/2: nutrient-replete, N/40: nitrogen-depleted, P/40: phosphorus-depleted). Vertical dashed line indicates the "harvest day" (the day that cells were collected for staining and experiments; notice that cell size among treatments was still the same).

Figure 1 presents the temporal changes in the *Heterocapsa* sp. batch cultures under the three nutrient conditions, in terms of both cell concentration and cell size (ESD, equivalent spherical diameter). The nutrient-depleted cultures (N/40 and P/40) had a shorter exponential phase and resulted in a lower yield. During the log phase, cell size did not vary among treatments, but after the sixth day, cells in the depleted media increased in size as they entered the static growth phase, whereas the f/2 cells became progressively smaller. Based on this trend, we opted to harvest the algal cultures for feeding experiments 5-6 days after their start up, when depleted treatments were still at the latest exponential phase and no difference in size had yet emerged. 

The stoichiometric composition of cells on the harvest day revealed substantial differences among treatments (Table 1). Compared to the f/2 cells, N-limited cells had a significantly lower nitrogen content (22% reduction) and higher C:N molar ratio (a difference of 2.6 units). No significant differences were detected either in the carbon and phosphorus cell content or in the C:P molar ratio between these two treatments. The N:P molar ratio was significantly higher (2.5 units) in the nutrient-replete culture.

**Table 1:** Cell properties of the three distinct *Heterocapsa* sp. cultures on the harvest day.

				**Student’s *t*-test (df=2)**	
**Cell properties**	**f/2**	**N/40**	**P/40**	**f/2 vs. N/40**	**f/2 vs. P/40**
ESD (μm)	14.2	13.9	14.1		
pg C cell^-1^	371 (6.9)	369 (1.9)	396 (9.1)	0.20^ns^	-2.21^ns^
pg N cell^-1^	49 (0.0)	38 (0.1)	47 (1.1)	76.37^***^	1.19^ns^
pg P cell^-1^	11 (0.3)	12 (0.2)	3 (0.1)	-1.70 ^ns^	30.98^***^
C:N	8.9 (0.2)	11.5 (0.1)	9.8 (0.0)	-11.63^**^	-4.50^*^
C:P	84.4 (2.6)	79.8 (1.7)	297.6 (9.7)	1.50 ^ns^	-21.25 ^**^
N:P	9.5 (0.2)	7.0 (0.1)	30.5 (1.0)	9.28^*^	-20.93^**^

Cell size (ESD: equivalent spherical diameter), elemental composition (C: carbon, N: nitrogen, P: phosphorus) and molar ratios of the dinoflagellate *Heterocapsa* sp. growing in three chosen nutrient conditions (f/2: nutrient-replete, N/40: nitrogen-depleted, P/40: phosphorus-depleted) and collected on the harvest day (Day 6, see text for details). Student’s *t*-tests were used to compare f/2 vs. N/40 cells and f/2 vs. P/40 cells (^*^: *p*<0.05, ^**^: *p*<0.01, ^***^: *p*<0.001, ^ns^.: not significant). Numbers in parentheses correspond to the standard error.

When the P-limited treatment was considered, the phosphorus cell content was significantly lower (by a factor of 3.7) than in the f/2 cells; no differences were detected in the carbon and nitrogen contents. Molar C:P and N:P ratios were both significantly higher for the P-limited cells (more than a 3-fold increase). The C:N ratio was slightly higher in the P/40 cells, but the difference was of marginal statistical significance (*p* = 0.046). Additional tests indicated that the staining and handling procedures had no significant influence on the elemental composition of the algae or their molar ratios (Table S1). 

We should note that we had expected a stronger difference between the elemental compositions of f/2 and N/40 cells than what was achieved. Analyses of the dissolved inorganic nutrients in the culture media showed, after taking into account the nitrogen content of the inoculum used, that the actual growing conditions at the start of the N-depleted cultures was N/18. In the case of phosphorus, the extra nutrient load was not relevant (actual value was P/37). Despite this issue, the *Heterocapsa* sp. cells used in our experiments had experienced a notable nutrient limitation in both depleted treatments when harvested (Figure S3).

### Single suspension feeding experiment

**Figure 2:**
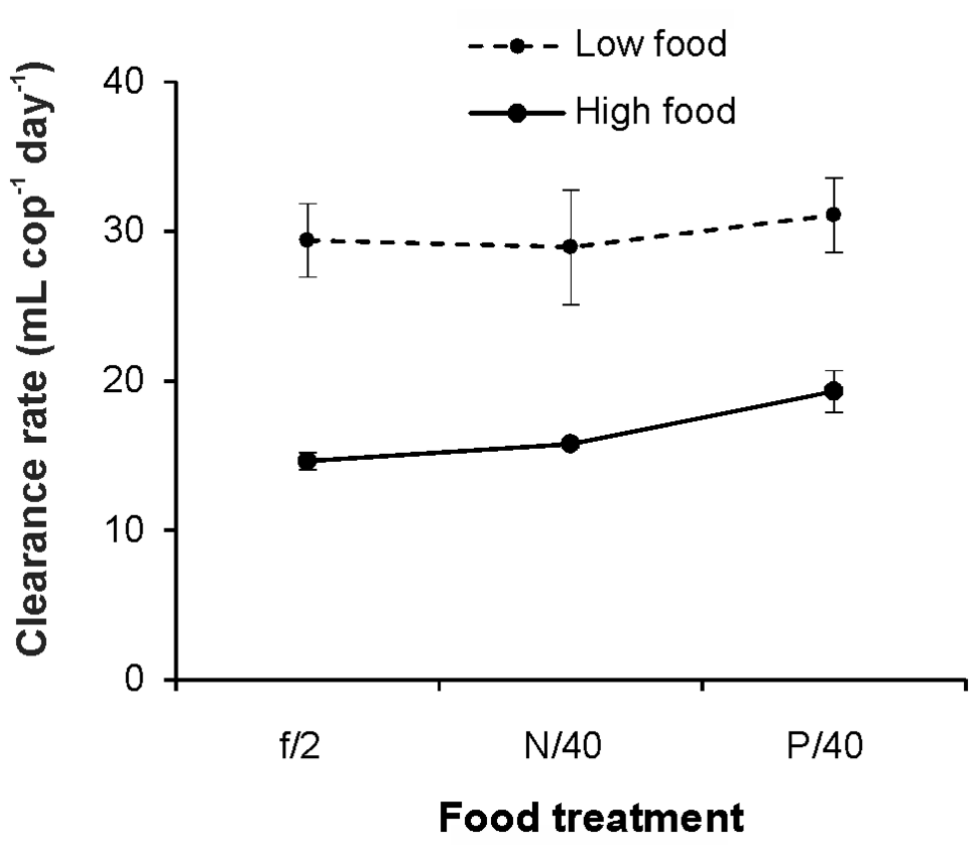
Clearance rates of *Acartia grani* on single algal suspensions. Average clearance rates (cells mL^-1^ cop^-1^) of *Acartia grani* on the three distinct single food suspensions (f/2, N/40 and P/40) at two food concentrations (low and high). Error bars indicate the standard error. The respective initial cell concentrations (cells mL^-1^) were as follows: f/2, low: 537; N/40, low: 505; P/40, low: 448; f/2, high: 2063; N/40, high: 2015; P/40, high: 1795.

**Figure 3:**
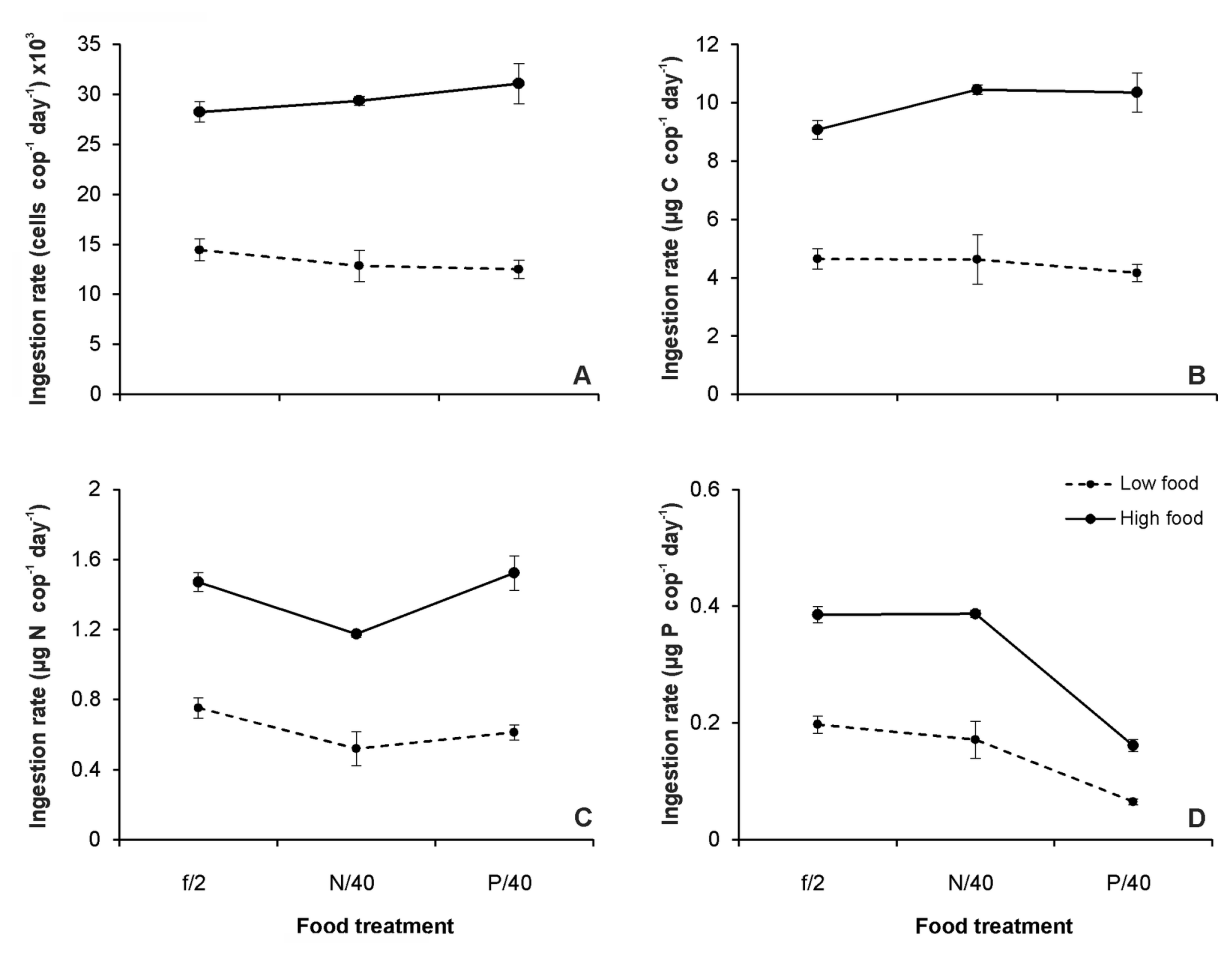
Ingestion rates of *Acartia grani* on single algal suspensions. Average ingestion rates of *Acartia grani* exposed to the three distinct single food suspensions (f/2, N/40 and P/40) at two food concentrations (low and high). Ingestion is expressed in different units: A) cells B) μg of carbon C C) μg of nitrogen N D) μg of phosphorus P. Error bars indicate the standard error.

In accordance with the results of our prior trials, the three food suspensions (f/2, N/40 and P/40) offered separately in the single suspension feeding experiments presented significant differences in cell stoichiometry (Table S2). A two-way ANOVA testing the dependence of the clearance rates of *A. grani* on food type and concentration showed that foods of all qualities were cleared at similar rates (*F*_2,12_=1.24, *p*>0.05), and only the food concentration had a significant effect (*F*_1,12_=53.70, *p*<0.001) (Figure 2); no significant interaction was observed between the two factors (*F*_2,12_=0.23, *p*>0.05). A comparison of ingestion rates did not find significant differences between treatments, indicating the lack of a compensatory feeding response under the nutrient-limited diets (N/40 and P/40) (Figure 3).

### Feeding selectivity experiments

**Figure 4:**
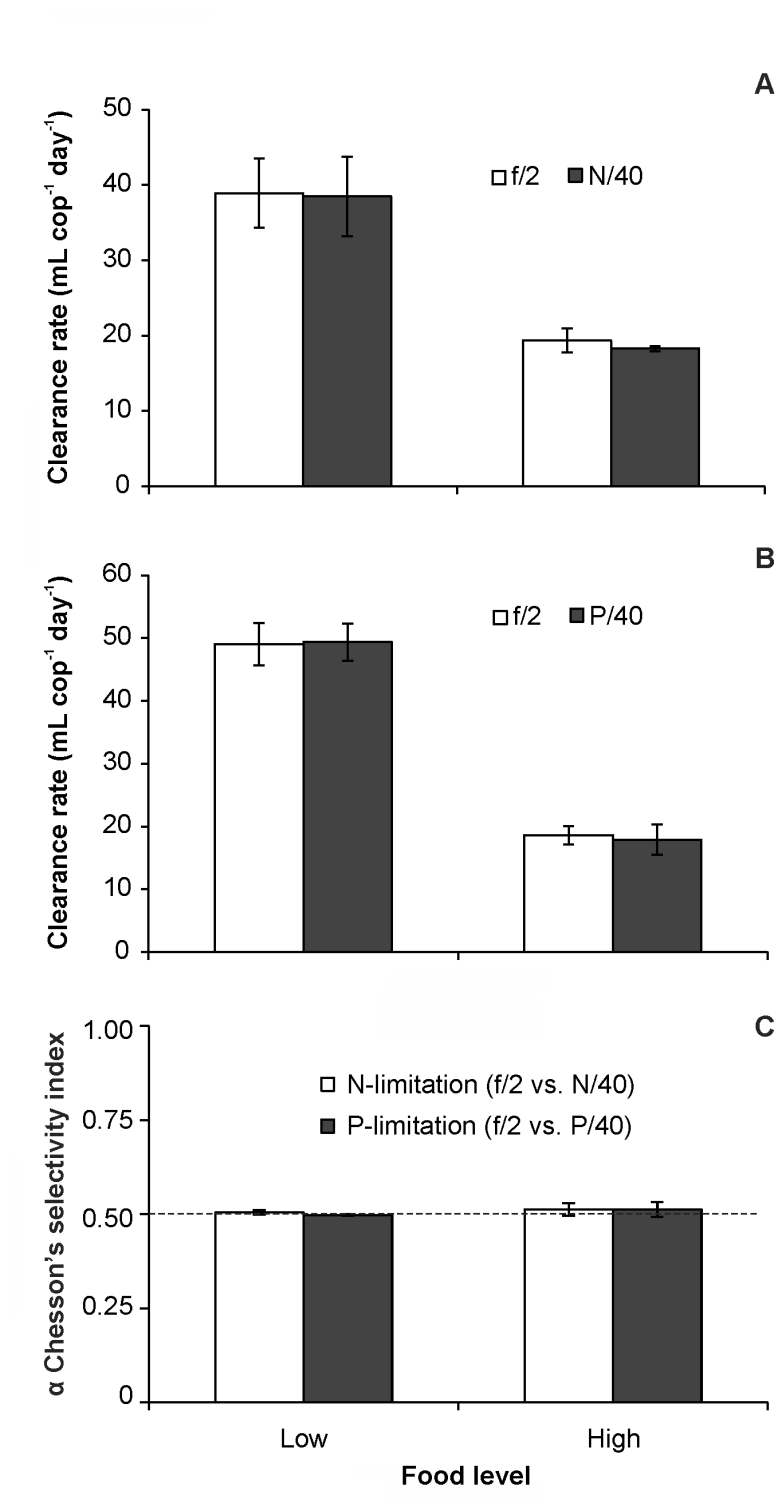
Copepod selective behaviour tested on mixed algal suspensions. Selective ability of *Acartia grani* when offered 1:1 mixtures of distinct *Heterocapsa* sp. cell types (replete vs. depleted ones) A) Average clearance rates (mL cop^-1^ day^-1^) under N-limitation (f/2 vs. N/40 cells) at low and high food concentrations (516±5 cells mL^-1^ and 2091±68 cells mL^-1^, respectively); B) Average clearance rates (mL cop^-1^ day^-1^) under P-limitation (f/2 vs. P/40 cells) at low and high food concentrations (447±14 cells mL^-1^ and 1758±11 cells mL^-1^, respectively); C) Chesson's selectivity index values for the f/2 *Heterocapsa* sp. cells under both N-limitation and P-limitation selectivity experiments. The dashed horizontal line represents a level of α where no selectivity is detected. Notice that as only two prey choices (f/2 vs. either N/40 or P/40 cells) were offered, only the selectivity index for f/2 cells is shown. Error bars indicate the standard error.

In both selectivity experiments, the proportion of replete and depleted cells in the suspension was ca. 1:1 (N-limitation: 47%:53%, P-limitation: 49%:51%, respectively). Tables S3 and S4 summarise the cell size and elemental composition of the prey used, which showed similar differences among treatments to those reported in previous tests (Tables 1 and S1). Regardless of food concentration, the clearance rates of *A. grani* on *Heterocapsa* sp. were similar between the distinct cell types in both the N-limitation (paired *t*-tests; low food: *t*=0.62, df=3, *p*>0.05; high food: *t*=0.79, df =3, *p*>0.05) and P-limitation experiments (paired *t*-tests; low food: *t*=-0.517, df=3, *p*>0.05; high food: *t*=0.53, df =3, *p*>0.05), indicating no signs of selection (Figure 4). This is further confirmed by the Chesson's selectivity index values, which also evidenced no selectivity under either N-limitation (paired *t*-tests; low food: *t*=0.86, df =3, *p* >0.05; high food: *t*=0.78, df =3, *p*>0.05) or P-limitation (paired *t*-tests; low food: *t*=-0.72, df =3, *p* >0.05; high food: *t*=0.64, df =3, *p* >0.05) (Figure 4).

### Egg production experiment

**Figure 5:**
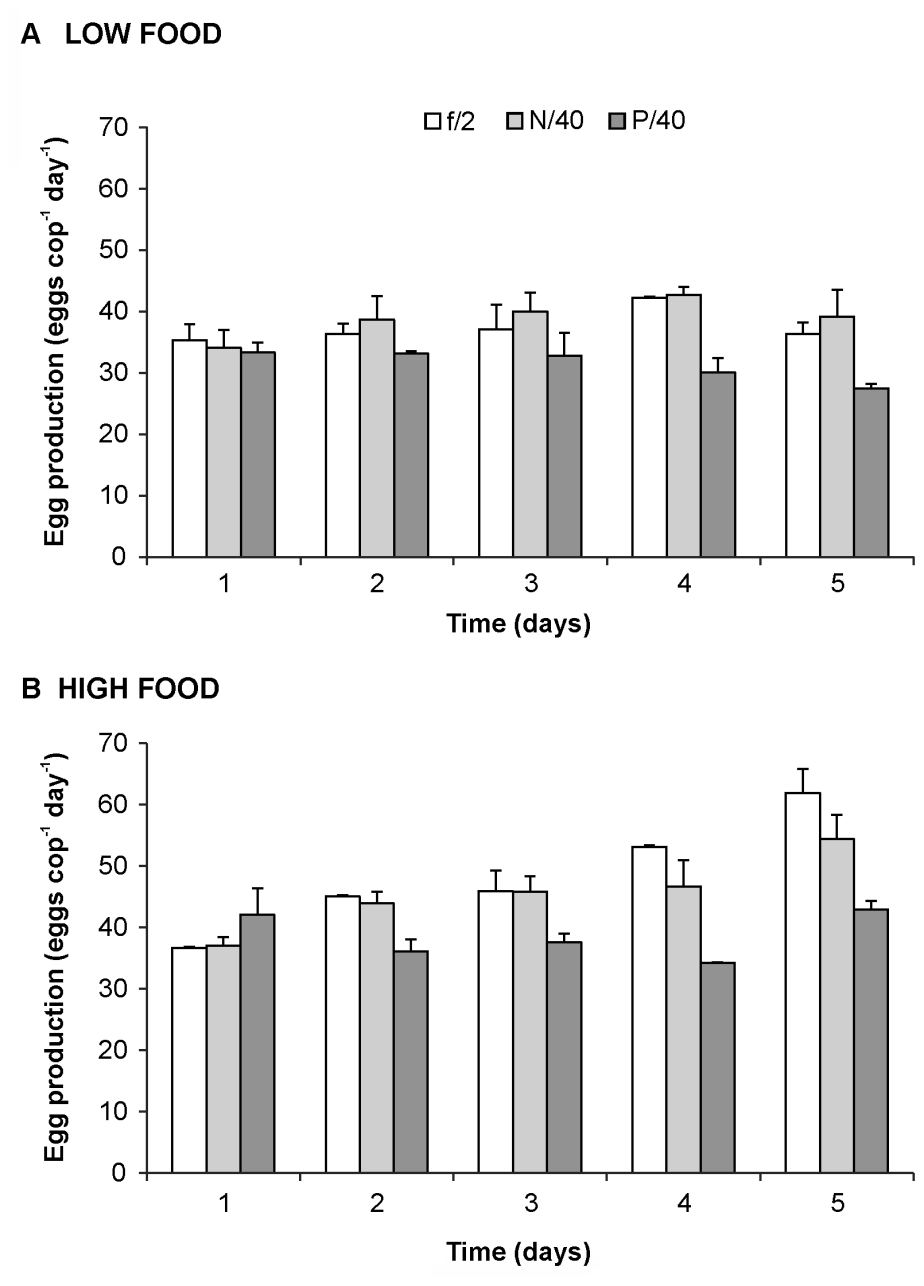
Copepod egg production under distinct *Heterocapsa* sp. diets. Temporal changes in egg production rates (eggs cop^-1^ day^-1^) of *Acartia grani* under three distinct diets of the dinoflagellate *Heterocapsa* sp. (f/2, N/40, P/40). Days 1-4: preconditioning, Day 5: main experiment. Food supply was provided at two food concentrations: A) low: 500 cells mL^-1^, and B) high: 2000 cells mL^-1^. Error bars indicate the standard error.

Although this experiment was designed to assess the effects of nutrient-limited algae after the preconditioning period (5th day), the egg counts conducted daily helped to better discern the trends. On the first day of acclimation, copepod egg production rates were similar among treatments (one-way ANOVA, *F*_5,6_=1.50, *p*>0.05), reflecting the previous common feeding history of the copepods. On the following days, copepod egg production differed according to the quality and quantity of the food supplied. From the second day onward, we always observed, at both food levels, lower egg production for the animals fed with P/40 *Heterocapsa* sp., although this difference was larger and statistically significant on the last two days (one-way ANOVAs; Figure 5). Specifically, at high food concentrations, copepods under a P/40 diet laid 25-30% and 30-35% less eggs than those in the f/2 treatment at low (one-way ANOVA and Tukey’s post-hoc tests; day 4^th^: *F*_2,3_=24.04, *p*<0.05; day 5^th^: *F*_2,11_=5.50, *p*<0.05) and high food levels (one-way ANOVA and Tukey’s post-hoc tests; day 4^th^: *F*_2,3_=13.81, *p*<0.05; day 5^th^: *F*_2,12_=6.66, *p*<0.05), respectively. Contrarily, the N/40 diet did not result in clear detrimental effects. At the low food concentration, the N/40 diet did not yield a significant difference in egg production compared to f/2 (one-way ANOVAs and Tukey’s post-hoc tests); at the high food concentration; on the last two days, copepods on the N/40 diet produced fewer eggs than those on the f/2 treatment, but the differences were not statistically significant (one-way ANOVAs and Tukey’s post-hoc tests). 

## Discussion

### Production of nutritionally distinct dinoflagellate types and discrimination when in mixtures

It is well documented that the physiological status and elemental/biochemical profile of algae are highly dependent on the growing conditions (e.g., light, nutrients; [49], [50]). Harvesting our *Heterocapsa* sp. cultures before the stationary phase ensured no difference in cell size or motility (visually checked) among cell types, and therefore the observed feeding patterns reflected only the copepod response to prey nutritional quality. The use of vital fluorochromes did not affect either the palatability or the biochemical composition of the labelled cells and allowed discrimination between mixtures of cells of different nutrient status, but morphologically undistinguishable. 

We assessed the quality of our food treatments through a stoichiometric approach, using molar ratios of inorganic nutrients as a proxy [51]. Despite elemental ratios being simple, bulk indicators of food quality [52], [53], the stoichiometric approach has proven successful in freshwater zooplankton research, e.g., [50], [54], [55]. Contrarily, nutritional studies of marine copepods have traditionally focused on biochemical compounds (e.g., protein, lipids), which may better define the quality of algae [30], [35], [37]. However, changes in the stoichiometry of algae do also reflect changes in the contents of biochemical compounds. In this regard, Klein Breteler et al. [37] reported that high algal C:N ratios under nutrient-limited conditions can be accompanied by lower contents of polyunsaturated fatty acids and sterols. Although we did not analyse the biochemical compositions of our distinct food types, Berdalet et al. [43] reported that the same *Heterocapsa* sp. strain growing under the N- and P- starved treatments of the current study may present, respectively, ca. 25% and 35% reductions in the protein cell content. Moreover, the outcome of the egg production experiments (as will be discussed later) also supports the substantial nutritional differences among the cell types used in our work.

### Copepod feeding and reproduction on the basis of food quality

Our unialgal grazing experiments indicated no influence of the elemental composition of prey on copepod ingestion rates, i.e., we found no evidence (at any food level) of either higher ingestion of the f/2 cells (in comparison with the nutrient-limited ones) or of compensatory overconsumption of the depleted ones. Reports of such responses to food quality in the marine copepod feeding literature are contradictory. Examples of higher rates of copepod feeding on better-quality algae (e.g., growing diatoms vs. senescent ones [7], [30]; diatoms with a lower C:N ratio and higher protein content [33]) and lower feeding rates under nitrogen-deficient algal mixtures [38] can be found in the literature. On the other hand, compensatory overconsumption has been reported under a nitrogen-depleted algal supply [32], [36] [56], and for stationary-phase diatoms [57]. In addition, poor algal nutritional quality may not necessarily result in effects on feeding [31], [32], [36]. The interpretation of such contrasting results among studies is rather puzzling. Although differences in experimental set-ups (e.g., algal growing conditions, copepod acclimation, incubation time etc.) do not allow straightforward comparisons, one could possibly discern contrasts even between studies on the same species [31], [33], [56]. It appears likely that the observed variability, at least to some extent, is related to species-specific physiological responses of both the predator and prey. For instance, Augustin and Boersma [36] reported that two congeneric copepod species may exhibit different feeding responses when offered identical diets; a single copepod species, moreover, may respond to nutrient limitation differently according to the prey offered [32], [38].

Previous studies have reported copepod selectivity (i.e., the selection of one prey type in the presence of alternative prey) on the basis of nutritional quality. For instance, *A. tonsa* may select food of better quality, maximising total protein ingestion, when supplied mixed suspensions of N-replete and N-depleted *Thalassiosira weissflogii* cells [33]. Similarly, the freshwater copepod *Diaptomus kenai* actively selected cells of a high N-content green algae species when offered simultaneously with a severely N-limited clone [34]. Such a capability to feed selectively as a function of the nutritional contents of prey would allow copepods to cope with the variability in food quality encountered in nature and to better satisfy their nutritional requirements for survival, development, growth and reproduction. Even under subtle food quality differences, copepods may feed selectively when food is abundant and non-selectively when food is scarce [18], [58], as predicted by optimal foraging theory. Contrary to these expectations, however, in our selectivity experiments, we found no discrimination among cells of distinct nutritional quality at any food level.

It is accepted that copepods might not sense very small prey individually, and therefore, selection by chemical cues might not be feasible [1]. However, we do not think that the discrepancy of our results with the above-mentioned studies [33], [34] is related to the size of the prey used in our experiments. In fact, the prey size used in Cowles et al. [33] (ca. diameter of 14 μm) was similar to ours, and that used in the work of Butler et al. [34] was even smaller (median size ca. 3 µm, their Figure 1). In both of these works, however, selection was observed. 

An alternative explanation for the lack of selectivity in our experiments could be that the stoichiometric difference among the cell types in our experiments might not reflect nutritional differences large enough to trigger a selectivity response. However, the nutritional conditions in our experiments were sufficiently different to affect (to some extent) egg production. Regarding phosphorus limitation, the egg production rates of *A. grani* quickly reacted (on the second day) to the phosphorus-limited diet and reached a 30% reduction in the last two days of exposure. Not as clear, however, were our observations under nitrogen limitation. Despite the 22% reduction in the nitrogen content of the N-limited cells (similar to the 25% lower protein content in the study of Berdalet et al. [43]), the N/40 diet did not have such a strong impact on the egg production of *A. grani*; a decrease in fecundity, though not significant, was observed only at the high food level (most likely due to a faster exhaustion of the limiting nutrient component at a higher egg production rate). 

We cannot exclude the possibility that the observed weak response to nitrogen-limitation might be the result of subtle cellular N-deficiency achieved in our experiments. For instance, if we estimate the theoretical threshold of the prey C:N ratio for nitrogen limitation in *Acartia* females, following the approach of Urabe and Watanabe [59] in *Daphnia*, we obtain a theoretical threshold (assuming copepod C:N of 4.5, [60], [61] and 30% carbon gross-growth efficiency) of ca. 15, much higher than the one we obtained in our N-limited conditions (C:N of ca. 11-12). However, the latter calculations assume that nitrogen is used with 100% efficiency, which is far from the 40% nitrogen efficiency for copepods shown experimentally [31] and predicted by model studies [62]; hence, this threshold is overestimated and very likely should be lower for *Acartia* and closer to the values we offered. In this regard, it is worth mentioning that moderate shifts in the C:N elemental ratio (similar to those in our experiments) are likely of more ecological relevance (as field deviations from the Redfield ratio are typically small, [31], [49], [63]) and have proven to be sufficient to significantly affect copepod egg production [31] and growth [32]. Actually, when the equation relating the prey C:N ratio to the egg production rate of *Acartia tonsa* reported by Kiørboe [31] is fitted with prey C:N values similar to the ones in our experiments, the low C:N diet (f/2) yields higher daily egg production than the high C:N diet (N/40), accounting for a increment of 8 eggs per copepod, similar to the difference (albeit not significant) found in our experiments on the fourth and fifth days. 

Prey selection on the basis of food quality is thought to be mediated through chemosensory input [64]; however, the overall mechanisms involved are not well understood. It has been suggested that the chemical gradient (phycosphere) around algal cells related to their cellular quality has the potential to transfer signals to copepods [65], [66]. Detection appears to occur at a shorter distance than previously believed [67], involving either gustation or tactile sensing, but the actual extracellular or cell-surface substances involved in chemical detection are still not well characterised, although amino acids are good candidates [68]. One may expect that large differences in prey C:N:P ratios (proxy of food quality) would result in distinct extracellular chemical gradients acting as signals to the predator. In the two available studies reporting selectivity as a function of prey quality in copepods [33], [34], the grazers were offered mixtures of clonal algae characterised by much higher differences in C:N molar ratios ([33]: ca. 7 units;[34]: ca. 9 units) compared to the ones offered in our study (ca. 3 units). It appears likely that although copepods are capable of responding selectively when offered food choices with strong nutritional differences, they may not be equally as capable of distinguishing between prey and feeding selectively when smaller or subtle differences are involved. However, this argument is in conflict with the fact that we still did not observe prey selection on the basis of prey nutritional quality in the case of the f/2 vs. P/40 mixture, when the differences in quality between cell types were much stronger (i.e., elemental ratios, egg production). We must conclude, therefore, that the lack of feeding selectivity by *Acartia grani* in our experiments, at least for the P-limitation, is not a consequence of too-small nutritional differences among cell types.

Other aspects may be taken into account to explain the discrepancy between our results and the two previous studies available [33,34]. Differences in elemental composition among algal types are primarily dependent on the growing conditions. Aside from growing conditions per se, taxon-specific characteristics may intensify some of the differences in elemental cell composition found in the literature among prey types of a certain species. In this regard, it is worth noting that when significant effects of distinct nutritional types of the same prey species on copepod vital rates have been reported, in most cases, the diatom genus *Thalassiosira* was offered as prey, e.g., [30], [31], [32]. Contrarily, when distinct nutritional types of non-diatom algae have been offered to copepods, either no significant difference in copepod vital rates has been detected [32], [35] or even the opposite response was obtained [36]. A taxon-specific algal physiological response to growing conditions has also been evidenced in other studies. For instance, Jones et al. [38] reported that even under the same N-limited growing conditions, dinoflagellates may have a lower C:N ratio (better quality) than diatoms; the latter study also reported higher copepod ingestion of N-sufficient compared to N-deficient prey in the case of diatoms, whereas dinoflagellates were consumed at similar rates regardless of their nutritional status. There appears to be an overall nutritional inferiority of diatoms relative to other phytoplankton, particularly dinoflagellates [35], [39], [69], although some studies suggest that other factors besides nutritional differences per se might help explain this pattern [70]. The importance of the choice of prey items for copepod experiments in which food quality is a limiting factor has been underlined, implying an unsuitability of the diatom *Thalassiosira* for these cases [35]. 

Laboratory experiments have provided strong evidence that copepods may respond selectively when offered two choices: “food” vs. “non-food items”, e.g., [14], [15] [18], and “food” vs. “harmful food” [18,24,25]. However, even when the prey items offered in mixtures differ substantially in terms of quality, the ability to select against non-food items may vary considerably depending on the copepod species, the acclimating conditions and the amount of food available [14], [15], [18]. Given the variability in response when coarse differences in quality are involved, it is not surprising that copepods may distinguish between “food” and “better food" only under strong elemental and biochemical differences between prey [33], [34]. On the contrary, the evolutionary pressure to discern toxic prey items must have been much stronger than the need to detect pure nutritional quality differences, and therefore active selection against “harmful food” [18,24,25] is anticipated, in which pre-ingestion chemosensory mechanisms [23], [25] or previous “trial-and-error” experiences [22] appear to be involved.

Our study questions the extent to which the capability of copepods to feed selectively on the basis of the chemical composition of the prey, as evidenced in previous laboratory studies [33,34], is applicable under natural conditions. In field studies with natural, mixed assemblages, it has not proved feasible to search for pure nutritional differences in prey, and typically factors such as size, motility and taxonomic composition are considered the major factors driving the feeding selectivity of copepods. Compared with the high diversity (taxa, size, motility) of potential prey items that copepods encounter in the oceans, the moderate or subtle expected differences due to purely nutritional quality are likely of minor relevance and it may be that only extreme differences are of importance. In addition, copepods in the oceans are in most cases food-limited, and from an evolutionary point of view it would be advantageous to be less selective when food is scarce; only under circumstances of high prey density due to patchiness might it pay off to choose the more nutritious food item and discard the lesser one. 

## Supporting Information

Figure S1 **Influence of algal staining and presence of thecas on copepod feeding.**
(DOCX)

Figure S2 **Functional feeding response of *Acartia grani* on the dinoflagellate *Heterocapsa* sp.**
(DOCX)

Figure S3 **Nutrient load of the *Heterocapsa* sp. cultures.**
(DOCX)

Table S1 **Cell properties of *Heterocapsa* sp. after the staining and handling process.**
(DOCX)

Table S2 **Cell properties of the *Heterocapsa* sp. types in single suspension experiment.**
(DOCX)

Table S3 **Cell properties of the *Heterocapsa* sp. types when offered in mixtures: f/2^st^ vs. N/40.**
(DOCX)

Table S4 **Cell properties of the *Heterocapsa* sp. types when offered in mixtures: f/2^st^ vs. P/40.**
(DOCX)

Text S1 **Effects of cell staining and the presence of thecas on grazing activity.**
(DOCX)

## References

[1] Price HJ, Paffenhöfer GA (1985) Perception of food availability by calanoid copepods. Arch Hydrobiol Beih Ergebn Limnol 21: 115–124.

[2] Price H (1988) Feeding mechanisms in marine and freshwater zooplankton. Bull Mar Sci 43: 327–343.

[3] Vanderploeg HA (1994) Zooplankton particle selection and feeding mechanisms. In: Wotton RS The biology of particles in aquatic systems. Boca Ratton: Lewis Publishers pp. 205–234.

[4] Paffenhöfer GA (1988) Feeding rates and behavior of zooplankton. Bull Mar Sci 43: 430–445.

[5] Paffenhöfer GA, Lewis KD (1990) Perceptive performance and feeding behavior of calanoid copepods. J Plankton Res 12: 933–946. doi:10.1093/plankt/12.5.933.

[6] Kiørboe T, Visser AW (1999) Predator and prey perception in copepods due to hydromechanical signals. Mar Ecol Prog Ser 179: 81–95. doi:10.3354/meps179081.

[7] Mullin MM (1963) Some factors affecting the feeding of marine copepods of the genus *Calanus* . Limnol Oceanogr 8: 239–250. doi:10.4319/lo.1963.8.2.0239.

[8] Frost BW (1972) Effects of size and concentration of food particles on the feeding behavior of the marine planktonic copepod *Calanus* *pacificus* . Limnol Oceanogr 17: 805–815. doi:10.4319/lo.1972.17.6.0805.

[9] Svensen C, Kiørboe T (2000) Remote prey detection in *Oithona* *similis*: hydromechanical versus chemical cues. J Plankton Res 22: 1155–1166. doi:10.1093/plankt/22.6.1155.

[10] Henriksen CI, Saiz E, Calbet A, Hansen BW (2007) Feeding activity and swimming patterns of *Acartia* *grani* and *Oithona* *davisae* nauplii in the presence of motile and non-motile prey. Mar Ecol Prog Ser 331: 119–129. doi:10.3354/meps331119.

[11] Yen J, Fields D (1992) Escape responses of *Acartia* *hudsonica* nauplii from the flow field of *Temora* *longicornis* . Arch Hydrobiol Beih Ergebn Limnol 36: 123–134.

[12] Broglio E, Johansson M, Jonsson PR (2001) Trophic interaction between copepods and ciliates: effects of prey swimming behavior on predation risk. Mar Ecol Prog Ser 220: 179–186. doi:10.3354/meps220179.

[13] Poulet SA, Marsot P (1978) Chemosensory grazing by marine calanoid copepods (arthropoda: crustacea). Science 200: 1403–1405. doi:10.1126/science.200.4348.1403. PubMed: 17736324. 17736324

[14] Donaghay PL, Small LF (1979) Food selection capabilities of the estuarine copepod *Acartia* *clausi* . Mar Biol 52: 137–146. doi:10.1007/BF00390421.

[15] Huntley ME, Barthel KG, Star JL (1983) Particle rejection by *Calanus* *pacificus*: discrimination between similarly sized particles. Mar Biol 74: 151–160. doi:10.1007/BF00413918.

[16] DeMott WR (1986) The role of taste in food selection by freshwater zooplankton. Oecologia 69: 334–340. doi:10.1007/BF00377053. 28311333

[17] DeMott WR (1988) Discrimination between algae and artificial particles by freshwater and marine copepods. Limnol Oceanogr 33: 397–408. doi:10.4319/lo.1988.33.3.0397.

[18] DeMott WR (1989) Optimal foraging theory as a predictor of chemically mediated food selection by suspension-feeding copepods. Limnol Oceanogr 34: 140–154. doi:10.4319/lo.1989.34.1.0140.

[19] Roman MR (1984) Utilization of detritus by the copepod, *Acartia* *tonsa* . Limnol Oceanogr 29: 949–959. doi:10.4319/lo.1984.29.5.0949.

[20] Paffenhöfer GA, Van Sant KB (1985) The feeding response of a marine planktonic copepod to quantity and quality of particles. Mar Ecol Prog Ser 27: 55–65. doi:10.3354/meps027055.

[21] Huntley M, Sykes P, Rohan S, Marin V (1986) Chemically-mediated rejection of dinoflagellate prey by the copepods *Calanus* *pacificus* and *Paracalanus* *parvus*: mechanism, occurrence and significance. Mar Ecol Prog Ser: 105–120.

[22] Uye S, Takamatsu K (1990) Feeding interactions between planktonic copepods and red-tide flagellates from Japanese coastal waters. Mar Ecol Prog Ser 59: 97–107. doi:10.3354/meps059097.

[23] Schultz M, Kiørboe T (2009) Active prey selection in two pelagic copepods feeding on potentially toxic and non-toxic dinoflagellates. J Plankton Res 31: 553–561. doi:10.1093/plankt/fbp010.

[24] Turriff N, Runge JA, Cembella AD (1995) Toxin accumulation and feeding behaviour of the planktonic copepod *Calanus* *finmarchicus* exposed to the red-tide dinoflagellate *Alexandrium* *excavatum* . Mar Biol 123: 55–64. doi:10.1007/BF00350323.

[25] Teegarden GJ (1999) Copepod grazing selection and particle discrimination on the basis of PSP toxin content. Mar Ecol Prog Ser 181: 163–176. doi:10.3354/meps181163.

[26] Colin SP, Dam HG (2003) Effects of the toxic dinoflagellate *Alexandrium* *fundyense* on the copepod *Acartia* *hudsonica*: a test of the mechanisms that reduce ingestion rates. Mar Ecol Prog Ser 248: 55–65. doi:10.3354/meps248055.

[27] Meyer-Harms B, Irigoien X, Head R, Harris R (1999) Selective feeding on natural phytoplankton by *Calanus* *finmarchicus* before, during, and after the 1997 spring bloom in the Norwegian Sea. Limnol Oceanogr 44: 154–165. doi:10.4319/lo.1999.44.1.0154.

[28] Saiz E, Calbet A (2011) Copepod feeding in the ocean: scaling patterns, composition of their diet and the bias of estimates due to microzooplankton grazing during incubations. Hydrobiologia 666: 181–196. doi:10.1007/s10750-010-0421-6.

[29] Meyer B, Irigoien X, Graeve M, Head RN, Harris R (2002) Feeding rates and selectivity among nauplii, copepodites and adult females of *Calanus* *finmarchicus* and *Calanus* *helgolandicus*. Helgol Mar. Resour 56: 169–176.

[30] Houde SEL, Roman MR (1987) Effects of food quality on the functional ingestion response of the copepod *Acartia* *tonsa* . Mar Ecol Prog Ser 40: 69–77. doi:10.3354/meps040069.

[31] Kiørboe T (1989) Phytoplankton growth rate and nitrogen content: implications for feeding and fecundity in a herbivorous copepod. Mar Ecol Prog Ser 55: 229–234. doi:10.3354/meps055229.

[32] Koski M, Klein Breteler W, Schogt N (1998) Effect of food quality on rate of growth and development of the pelagic copepod *Pseudocalanus* *elongatus* (Copepoda, Calanoida). Mar Ecol Prog Ser 170: 169–187. doi:10.3354/meps170169.

[33] Cowles TJ, Olson RJ, Chisholm SW (1988) Food selection by copepods: discrimination on the basis of food quality. Mar Biol 100: 41–49. doi:10.1007/BF00392953.

[34] Butler NM, Suttle CA, Neill WE (1989) Discrimination by freshwater zooplankton between single algal cells differing in nutritional status. Oecologia 78: 368–372. doi:10.1007/BF00379111. 28312583

[35] Jónasdóttir S (1994) Effects of food quality on the reproductive success of *Acartia* *tonsa* and *Acartia* *hudsonica*: laboratory observations. Mar Biol 121: 67–81. doi:10.1007/BF00349475.

[36] Augustin CB, Boersma M (2006) Effects of nitrogen stressed algae on different *Acartia* species. J Plankton Res 28: 429–436. doi:10.1093/plankt/fbi131.

[37] Klein Breteler WCM, Schogt N, Rampen S (2005) Effect of diatom nutrient limitation on copepod development: the role of essential lipids. Mar Ecol Prog Ser 291: 125–133. doi:10.3354/meps291125.

[38] Jones RH, Flynn KJ, Anderson TR (2002) Effect of food quality on carbon and nitrogen growth efficiency in the copepod *Acartia* *tonsa* . Mar Ecol Prog Ser 235: 147–156. doi:10.3354/meps235147.

[39] Jones RH, Flynn KJ (2005) Nutritional status and diet composition affect the value of diatoms as copepod prey. Science 307: 1457–1459. doi:10.1126/science.1107767. PubMed: 15746424. 15746424

[40] Benitez-Nelson CR (2000) The biogeochemical cycling of phosphorus in marine systems. Earth Sci Rev 51: 109–135. doi:10.1016/S0012-8252(00)00018-0.

[41] Thingstad TF, Krom MD, Mantoura RFC, Flaten GAF, Groom S et al. (2005) Nature of Phosphorus Limitation in the Ultraoligotrophic Eastern Mediterranean. Science 309: 1068–1071. doi:10.1126/science.1112632. PubMed: 16099984. 16099984

[42] Elser JJ, Bracken MES, Cleland EE, Gruner DS, Harpole WS et al. (2007) Global analysis of nitrogen and phosphorus limitation of primary producers in freshwater, marine and terrestrial ecosystems. Ecol Lett 10: 1135–1142. doi:10.1111/j.1461-0248.2007.01113.x. PubMed: 17922835. 17922835

[43] Berdalet E, Latasa M, Estrada M (1994) Effects of nitrogen and phosphorus starvation on nucleic acid and protein content of *Heterocapsa* sp. J Plankton Res 16: 303–316. doi:10.1093/plankt/16.4.303.

[44] Latasa M, Berdalet E (1994) Effect of nitrogen or phosphorus starvation on pigment composition of cultured *Heterocapsa* sp. J Plankton Res 16: 83–94. doi:10.1093/plankt/16.1.83.

[45] Guillard R (1975) Culture of phytoplankton for feeding marine invertebrates. In: Smith WL Chanley M Culture of marine invertebrate animals. New York, USA: Plenum Press pp. 26–60.

[46] Berdalet E, Latasa M, Estrada M (1992) Variations in biochemical parameters of *Heterocapsa* sp. and *Olisthodiscus* *luteus* grown in 12:12 light: dark cycles I. Cell cycle and nucleic acid composition. Hydrobiologia 238: 139–147. doi:10.1007/BF00048782.

[47] Grasshoff K, Kremling K, Ehrhardt M (1999) Methods of seawater analysis. 3rd ed. Weinheim, New York, Chiester, Brisbane, Singopore, Toronto: Wiley-VCH . 577 p

[48] Chesson J (1983) The estimation and analysis of preference and its relatioship to foraging models. Ecology 64: 1297–1304. doi:10.2307/1937838.

[49] Geider RJ, La Roche J (2002) Redfield revisited: variability of C:N:P in marine microalgae and its biochemical basis. Eur J Phycol 37: 1–17. doi:10.1017/S0967026201003456.

[50] Hessen DO, Færøvig PJ, Andersen T (2002) Light, nutrients and P:C ratios in algae: grazer performance related to food quality and quantity. Ecology 83: 1886–1898. Available online at: doi:10.1890/0012-9658(2002)083[1886:LNAPCR]2.0.CO;2.

[51] Sterner RW, Elser JJ (2002) Ecological stoichiometry: The biology of elements from molecules to the biosphere. Princeton: Princeton University Press. 439 pp.

[52] Tang KW, Dam HG (1999) Limitation of zooplankton production: beyond stoichiometry. Oikos 84: 537–542. doi:10.2307/3546434.

[53] Anderson TR, Pond DW (2000) Stoichiometric theory extended to micronutrients: comparison of the roles of essential fatty acids, carbon, and nitrogen in the nutrition of marine copepods. Limnol Oceanogr 45: 1162–1167. doi:10.4319/lo.2000.45.5.1162.

[54] Sterner RW, Hessen DO (1994) Algal nutrient limitation and the nutrition of aquatic herbivores. Annu Rev Ecol Syst 25: 1–29. doi:10.1146/annurev.es.25.110194.000245.

[55] Elser JJ, Hayakawa K, Urabe J (2001) Nutrient limitation reduces food quality for zooplankton : *Daphnia* response to seston phosphorus enrichment. Ecology 82: 898–903. Available online at: doi:10.1890/0012-9658(2001)082[0898:NLRFQF]2.0.CO;2.

[56] Siuda ANS, Dam HG (2010) Effects of omnivory and predator-prey elemental stoichiometry on planktonic trophic interactions. Limnol Oceanogr 55: 2107–2116. doi:10.4319/lo.2010.55.5.2107.

[57] Barofsky A, Simonelli P, Vidoudez C, Troedsson C, Nejstgaard JC et al. (2010) Growth phase of the diatom *Skeletonema* *marinoi* influences the metabolic profile of the cells and the selective feeding of the copepod *Calanus* spp. J Plankton Res 32: 263–272. doi:10.1093/plankt/fbp121.

[58] DeMott WR (1995) Optimal foraging by a suspension-feeding copepod: responses to short-term and seasonal variation in food resources. Oecologia 103: 230–240. doi:10.1007/BF00329085. 28306778

[59] Urabe J, Watanabe Y (1992) Possibility of N or P limitation for planktonic cladocerans: An experimental test. Limnol Oceanogr 37: 244–251. doi:10.4319/lo.1992.37.2.0244.

[60] Saba GK, Steinberg DK, Bronk DA (2009) Effects of diet on release of dissolved organic and inorganic nutrients by the copepod *Acartia* *tonsa* . Mar Ecol Prog Ser 386: 147–161. doi:10.3354/meps08070.

[61] Walve J, Larsson U (1999) Carbon , nitrogen and phosphorus stoichiometry of crustacean zooplankton in the Baltic Sea: implications for nutrient recycling. 21: 2309–2321.

[62] Kuijper LDJ, Anderson TR, Kooijman ALM (2004) C and N gross growth efficiencies of copepod egg production studied using a Dynamic Energy Budget model. J Plankton Res 26: 213–226. doi:10.1093/plankt/fbh020.

[63] Frigstad H, Andersen T, Hessen DO, Naustvoll L-J, Johnsen TM et al. (2011) Seasonal variation in marine C:N:P stoichiometry: can the composition of seston explain stable Redfield ratios? Biogeosciences 8: 2917–2933. doi:10.5194/bg-8-2917-2011.

[64] Friedman MM, Strickler JR (1975) Chemoreceptors and feeding in calanoid copepods (Arthropoda: Crustacea). Proc Natl Acad Sci U S A 72: 4185–4188. doi:10.1073/pnas.72.10.4185. PubMed: 1060099. 1060099 PMC433165

[65] Andrews JC (1983) Deformation of the active space in the low Reynolds number feeding current of calanoid copepods. Can J Fish Aquat Sci 40: 1293–1302. doi:10.1139/f83-147.

[66] Moore PA, Fields DM, Yen J (1999) Physical constraints of chemoreception in foraging copepods. Limnol Oceanogr 44: 166–177. doi:10.4319/lo.1999.44.1.0166.

[67] Tiselius P, Saiz E, Kiørboe T (2013) Sensory capabilities and food capture of two small copepods, *Paracalanus* *parvus* and *Pseudocalanus* sp. Limnol Oceanogr 58: 1657–1666. doi:10.4319/lo.2013.58.5.1657.

[68] Gill C, Poulet S (1988) Responses of copepods to dissolved free amino acids. Mar Ecol Prog Ser 43: 269–276. doi:10.3354/meps043269.

[69] Kleppel G (1993) On the diets of calanoid copepods. Mar Ecol Prog Ser 99: 183–195. doi:10.3354/meps099183.

[70] Dutz J, Koski M, Jónasdóttir SH (2008) Copepod reproduction is unaffected by diatom aldehydes or lipid composition. Limnol Oceanogr 53: 225–235. doi:10.4319/lo.2008.53.1.0225.

