# Supplementary material for: Copepod Foraging on the Basis of Food Nutritional Quality: Can Copepods Really Choose?
Source: PLoS One. 2013 Dec 26;8(12):e84742. doi: 10.1371/journal.pone.0084742 (PMC3873455; doi:10.1371/journal.pone.0084742)
Supplement: Figure S1 — Influence of algal staining and presence of thecas on copepod feeding. (DOCX) [file pone.0084742.s001.docx]

**
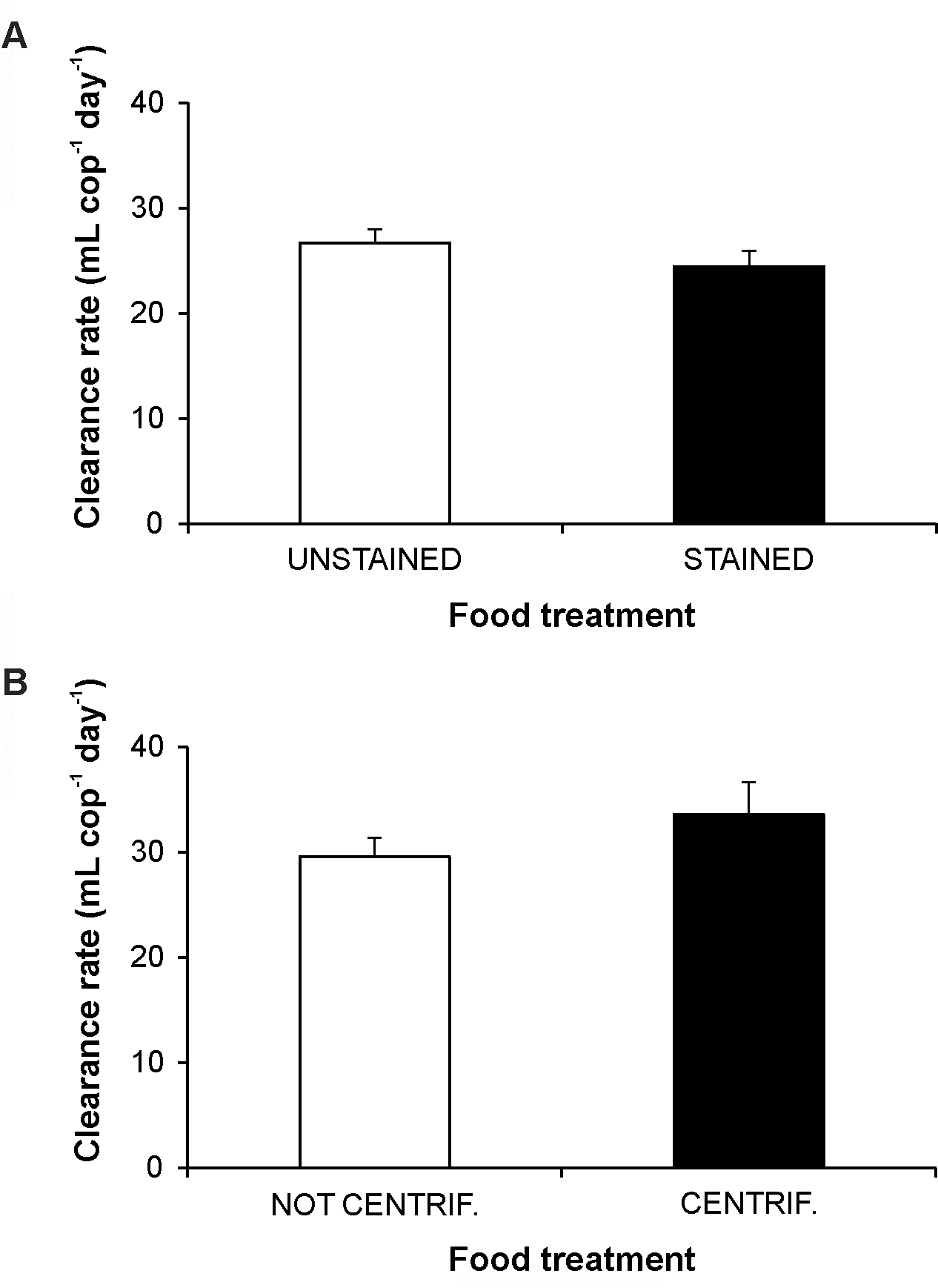
**

**Figure S1**. **Influence of algal staining and presence of thecas on copepod feeding.**

Feeding response of *Acartia grani* on *Heterocapsa* sp. cells in the experiments testing the influence of staining procedure (A) and the presence of free thecas in the food suspension (B). Average clearance rates (mL cop^-1^ day^-1^) are provided for copepods feeding on stained and unstained cells and feeding on cells that had or had not experienced the centrifugation treatment. Error bars represent the standard error.
