# Supplementary material for: Copepod Foraging on the Basis of Food Nutritional Quality: Can Copepods Really Choose?
Source: PLoS One. 2013 Dec 26;8(12):e84742. doi: 10.1371/journal.pone.0084742 (PMC3873455; doi:10.1371/journal.pone.0084742)
Supplement: Figure S2 — Functional feeding response of Acartia grani on the dinoflagellate Heterocapsa sp. (DOCX) [file pone.0084742.s002.docx]

**
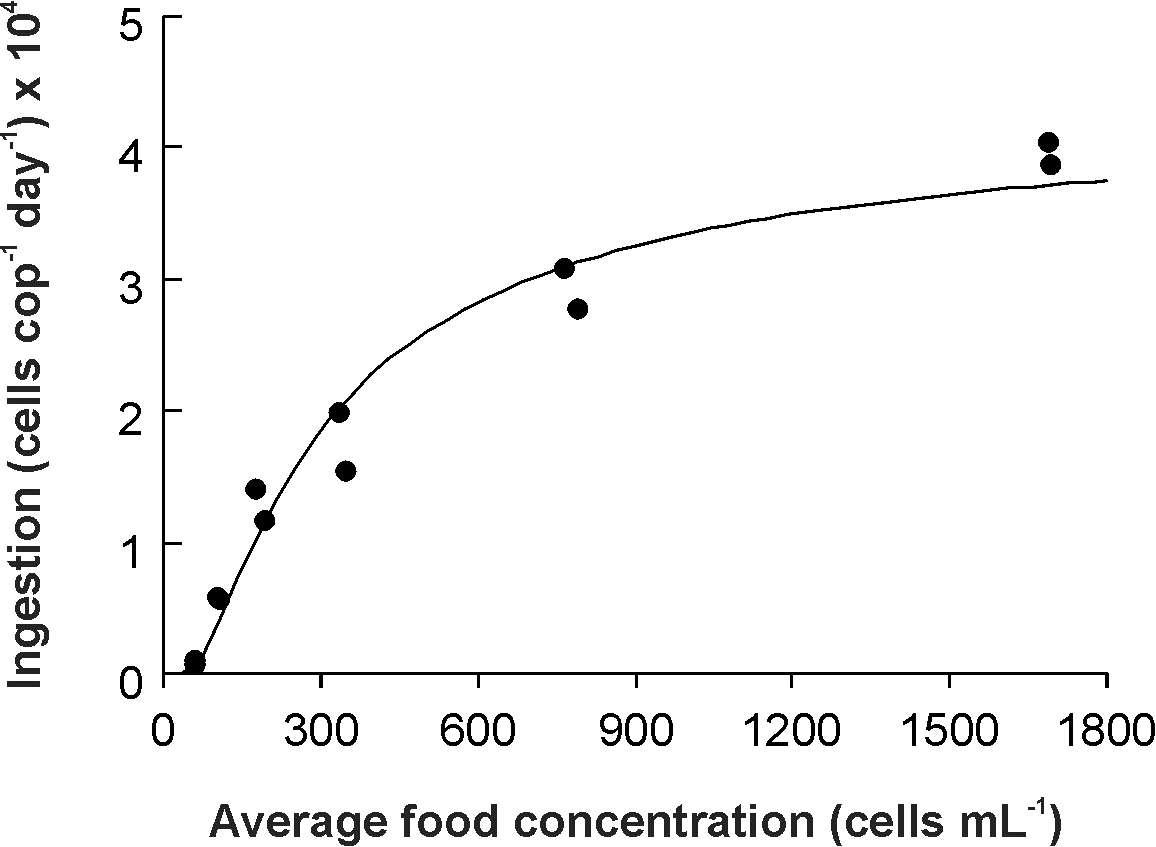
**

**Figure S2. Functional feeding response of *Acartia grani* on the dinoflagellate *Heterocapsa* sp.**

Ingestion rates (cells cop^-1^ day^-1^) of *Acartia grani* on the dinoflagellate *Heterocapsa* sp. as a function of the average concentration of *Heterocapsa* cells. A sigmoid model was fitted to the data, I=43208*exp^(-255/C)^, where I corresponds to the ingestion rates and C the average food concentration.
