# Supplementary material for: Copepod Foraging on the Basis of Food Nutritional Quality: Can Copepods Really Choose?
Source: PLoS One. 2013 Dec 26;8(12):e84742. doi: 10.1371/journal.pone.0084742 (PMC3873455; doi:10.1371/journal.pone.0084742)
Supplement: Figure S3 — Nutrient load of the Heterocapsa sp. cultures. (DOCX) [file pone.0084742.s003.docx]

**
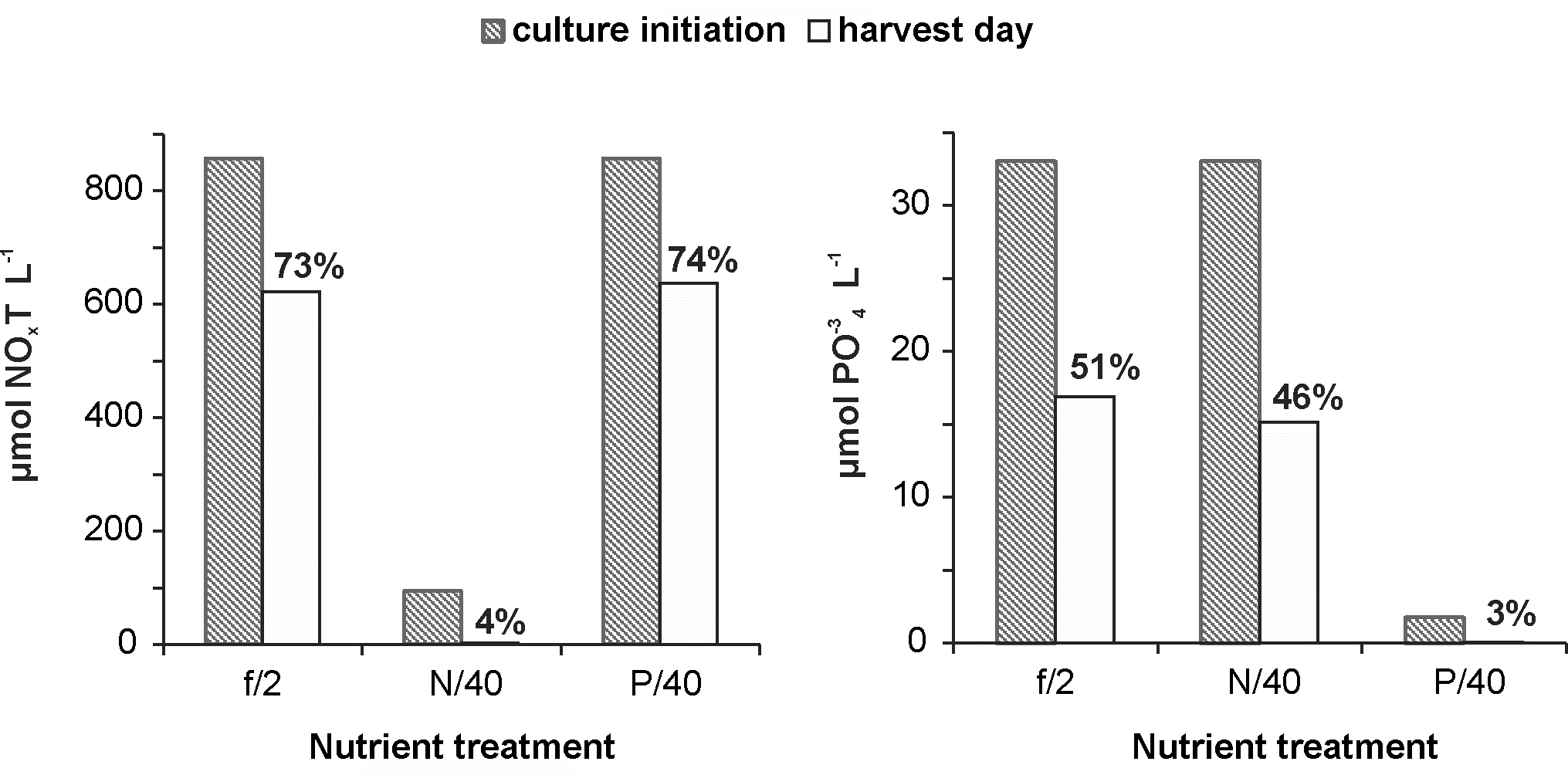
**

**Figure S3. Nutrient load of the *Heterocapsa* sp. cultures.**

Dissolved inorganic nutrient load (nitrate + nitrite, phosphate) of the culture media in the three growing conditions of the dinoflagellate *Heterocapsa* sp. at the time of the initiation of the cultures and on the harvest day. Percentages show the remaining nutrient concentration in relation to the initial load (f/2: nutrient-replete, N/40: nitrogen-depleted, P/40: phosphorus-depleted).
