# Supplementary material for: Copepod Foraging on the Basis of Food Nutritional Quality: Can Copepods Really Choose?
Source: PLoS One. 2013 Dec 26;8(12):e84742. doi: 10.1371/journal.pone.0084742 (PMC3873455; doi:10.1371/journal.pone.0084742)
Supplement: Table S1 — Cell properties of Heterocapsa sp. after the staining and handling process. (DOCX) [file pone.0084742.s004.docx]

**Table S1. Cell properties of *Heterocapsa* sp. after the staining and handling process**

|  |  |  |  |  | **Student’s *t*-test (df=2)** | | |
| --- | --- | --- | --- | --- | --- | --- | --- |
| **Cell properties** | **f/2^st^** | **f/2^unst^** | **N/40** | **P/40** | **f/2^st^ vs. f/2^unst^** | **f/2 ^st^ vs. N/40** | **f/2 ^st^ vs. P/40** |
| ESD (μm) | 14.1 | 13.9 | 13.7 | 14.1 |  |  |  |
| pg C cell^-1^ | 368 (2.9) | 352 (21.8) | 374 (21.2) | 414 (18.3) | 0.73^ns^ | -0.30 ^ns^ | -2.51^ns^ |
| pg N cell^-1^ | 47 (0.2) | 45 (0.7) | 36 (0.1) | 47 (0.1) | -3.01^ns^ | 53.00^***^ | 3.61^ns^ |
| pg P cell^-1^ | 10 (0.5) | 10 (0.6) | 10 (0.2) | 3 (0.2) | -0.24^ns^ | 0.09 ^ns^ | 12.05^**^ |
| C:N | 9.1 (0.0) | 9.1 (0.4) | 12.2 (0.6) | 10.4 (0.5) | 0.13^ns^ | -4.76^*^ | -2.69^ns^ |
| C:P | 92.9 (4.8) | 90.6 (8.0) | 95.0 (5.7) | 332.4 (25.5) | 0.25^ns^ | -0.28 ^ns^ | -9.23^*^ |
| N:P | 10.2 (0.5) | 9.9 (0.6) | 7.8 (0.2) | 32.0 (2.0) | 0.37^ns^ | 4.43^*^ | -10.50^**^ |

Cell size (ESD: equivalent spherical diameter), elemental composition (C: carbon, N: nitrogen, P: phosphorus) and molar elemental ratios of the dinoflagellate *Heterocapsa* sp. collected on the harvest day and either stained with Cell Blue Tracker (f/2^st^ cells) or treated with the same handling procedure (centrifuging, overnight recovering) but without addition of the fluorochrome (f/2^unst^, P/40 and N/40 cells). Student’s t-tests were used to compare f/2^st^ vs. f/2 cells, f/2^st^ vs. N/40 cells, and f/2^st^ vs. P/40 cells (df=2 for all comparisons; ^*^: *p*<0.05, ^**^: *p*<0.01, ^***^: *p*<0.001, ^ns^: not significant). Numbers in parentheses correspond to the standard error.
