# Supplementary material for: Copepod Foraging on the Basis of Food Nutritional Quality: Can Copepods Really Choose?
Source: PLoS One. 2013 Dec 26;8(12):e84742. doi: 10.1371/journal.pone.0084742 (PMC3873455; doi:10.1371/journal.pone.0084742)
Supplement: Table S2 — Cell properties of the Heterocapsa sp. types in single suspension experiment. (DOCX) [file pone.0084742.s005.docx]

**Table S2. Cell properties of the *Heterocapsa* sp. types in single suspension experiment**

|  |  |  |  |  | **Student’s *t*-test (df=2)** | |
| --- | --- | --- | --- | --- | --- | --- |
| **Cell properties** | **f/2** | **N/40** | **P/40** | ***F***_2,3_ | **f/2 vs. N/40** | **f/2 vs. P/40** |
| ESD (μm) | 14.2 | 13.7 | 13.9 |  |  |  |
| pg C cell^-1^ | 321 (9.2) | 356 (0.3) | 333 (7.5) | 6.56 ^ns^ |  |  |
| pg N cell^-1^ | 52 (0.3)^a^ | 40 (1.2)^b^ | 49 (2.9)^a,b^ | 12.57^*^ |  |  |
| pg P cell^-1^ | 14 (0.2)^a^ | 13 (0.1)^a^ | 5 (0.2)^b^ | 935.21^***^ |  |  |
| C:N | 7.2 (0.3)^a^ | 10.4 (0.3)^b^ | 7.9 (0.3)^a^ | 37.53^**^ |  |  |
| C:P | 60.8 (1.9) | 69.8 (0.5) | 166.2 (7.6) |  | -4.50^*^ | -13.47^**^ |
| N:P | 8.5 (0.1) | 6.7 (0.2) | 21.1 (1.3) |  | 7.21^*^ | -8.53^*^ |

Cell size (ESD: equivalent spherical diameter), elemental composition (C: carbon, N: nitrogen, P: phosphorus) and molar elemental ratios of the distinct *Heterocapsa* sp. cultures offered during the single source feeding experiment. Differences among treatments were assessed with one-way ANOVA and Tukey’s post-hoc tests (*F*: ANOVA statistics, ^*^: *p*<0.05, ^**^: *p*<0.01, ^***^: *p*<0.001, ^ns^: not significant). Paired comparisons of C:P and N:P ratios conducted with Student’s *t*-tests (f/2 vs. N/40 and f/2 vs. P/40 cells). Numbers in parentheses correspond to the standard error and superscripts indicate the homogenous groups according to Tukey’s post-hoc test.
