# Supplementary material for: Copepod Foraging on the Basis of Food Nutritional Quality: Can Copepods Really Choose?
Source: PLoS One. 2013 Dec 26;8(12):e84742. doi: 10.1371/journal.pone.0084742 (PMC3873455; doi:10.1371/journal.pone.0084742)
Supplement: Table S3 — Cell properties of the Heterocapsa sp. types when offered in mixtures: f/2st vs. N/40. (DOCX) [file pone.0084742.s006.docx]

**Table S3. Cell properties of the *Heterocapsa* sp. types when offered in mixtures: f/2^st^ vs. N/40**

| **Cell properties** | **f/2^st^** | **N/40** | **Student’s *t*-test** |
| --- | --- | --- | --- |
| ESD (μm) | 13.3 | 13.7 |  |
| pg C cell^-1^ | 322 (0.9) | 347 (7.5) | -3.28^ns^ |
| pg N cell^-1^ | 44 (0.6) | 34 (1.7) | 5.68^*^ |
| pg P cell^-1^ | 11 (0.3) | 10 (0.1) | 1.17 ^ns^ |
| C:N | 8.5 (0.1) | 11.9 (0.3) | -9.35^*^ |
| C:P | 78.0 (2.0) | 86.9 (1.9) | -3.11 ^ns^ |
| N:P | 9.2 (0.3) | 7.3 (0.4) | 4.19 ^ns^ |

Cell size (ESD: equivalent spherical diameter), elemental composition (C: carbon, N: nitrogen, P: phosphorus) and molar elemental ratios of the distinct *Heterocapsa* sp. cultures offered in grazing experiments with mixtures of nutrient-replete (f/2^st^: f/2 cells stained with fluorochrome) and nutrient-depleted (N/40) prey. Paired comparisons for f/2^st^ vs. N/40 were conducted with Student’s *t*-test (df=2; ^*^: *p*<0.05, ^**^: *p*<0.01, ^***^: *p*<0.001, ^ns^: not significant). Numbers in parentheses correspond to the standard error.
