# Supplementary material for: Copepod Foraging on the Basis of Food Nutritional Quality: Can Copepods Really Choose?
Source: PLoS One. 2013 Dec 26;8(12):e84742. doi: 10.1371/journal.pone.0084742 (PMC3873455; doi:10.1371/journal.pone.0084742)
Supplement: Table S4 — Cell properties of the Heterocapsa sp. types when offered in mixtures: f/2st vs. P/40. (DOCX) [file pone.0084742.s007.docx]

**Table S4. Cell properties of the *Heterocapsa* sp. types when offered in mixtures: f/2^st^ vs. P/40**

| **Cell properties** | **f/2^st^** | **P/40** | **Student’s *t*-test** |
| --- | --- | --- | --- |
| ESD (μm) | 14.1 | 13.8 |  |
| pg C cell^-1^ | 326 (5.5) | 348 (1.8) | -3.79^ns^ |
| pg N cell^-1^ | 42 (3.3) | 42 (0.0) | 1.24^ns^ |
| pg P cell^-1^ | 12 (0.2) | 5 (0.7) | 10.27^**^ |
| C:N | 9.0 (0.1) | 9.9 (0.1) | -7.13^*^ |
| C:P | 70.6 (1.6) | 181.3 (24.1) | -4.59^*^ |
| N:P | 7.9 (0.1) | 18.7 (2.5) | -4.37^*^ |

Cell size (ESD: equivalent spherical diameter), elemental composition (C: carbon, N: nitrogen, P: phosphorus) and molar elemental ratios of the distinct *Heterocapsa* sp. cultures offered in grazing experiments with mixtures of nutrient-replete (f/2^st^: f/2 cells stained with fluorochrome) and nutrient-depleted (P/40) prey. Paired comparisons for f/2^st^ vs. P/40 were conducted with Student’s *t*-test (df=2; ^*^: *p*<0.05, ^**^: *p*<0.01, ^***^: *p*<0.001, ^ns^: not significant). Numbers in parentheses correspond to the standard error.
