# Supplementary material for: Copepod Foraging on the Basis of Food Nutritional Quality: Can Copepods Really Choose?
Source: PLoS One. 2013 Dec 26;8(12):e84742. doi: 10.1371/journal.pone.0084742 (PMC3873455; doi:10.1371/journal.pone.0084742)
Supplement: Text S1 — Effects of cell staining and the presence of thecas on grazing activity. (DOCX) [file pone.0084742.s008.docx]

**Text S1*: Effects of cell staining and the presence of thecas on grazing activity***

To examine the influence of cell staining on the grazing activity of *Acartia grani*, two food suspensions (ca. 1200 cells mL^-1^) were prepared from f/2-medium batch cultures of *Heterocapsa* sp.: one was made with cells stained with fluorochrome and the other with cells handled exactly the same way but without the fluorochrome addition. For each experimental treatment, eight 625-mL Pyrex screw-cap bottles were set up: twelve female copepods per bottle were added to three of them (3 x Cop), whereas the other five bottles served as initial (2 x Init) and control (3 x Contr) bottles. Initial bottles were immediately sampled (50 mL samples) and preserved in 2% Lugol’s solution, and the rest were incubated on a slowly rotating plankton wheel (0.2 rpm) for 24 h at 18°C. At the end of the incubation, the contents of all bottles were carefully poured through a 200-µm mesh, the actual number and condition of copepods were checked (mortality was negligible), and 50-mL samples were preserved as above. To test the influence of the free thecas in the food suspension, two different *Heterocapsa* sp. suspensions (ca. 500 cells mL^-1^) were prepared, one consisting of centrifuged cells (in this case, after centrifugation the pellet was re-suspended in its own initial medium and the cells were left to recover) and the other consisting of cells that had not been centrifuged. We applied the same protocol for setting up (number of replicates, but 6 animals per Cop bottle) and taking down as described above. In both experiments, cell concentration was determined by inverted microscopy, counting at least 400-500 cells. The clearance and ingestion rates as well as average food concentrations were computed for each treatment according to the equation of Frost [1].

Figure S1 shows the results of the experiments conducted to assess the influence of cell staining and the presence of free thecas in the food suspension (EXP_stain_ and EXP_thecas_, respectively) on copepod feeding (clearance) rates. We found no influence of the staining process on the palatability of *Heterocapsa* sp., as both unstained and stained cells were cleared at equal rates (*t-*test=-1.16, df=4, *p*>0.05). Similarly, centrifugation of the cells and the subsequent release of free thecas in the suspension did not have an influence on copepod feeding activity, as *A. grani* cleared cells in both food treatments at similar rates (*t-*test = 1.02, df =4, *p* >0.05). In each experiment, initial cell concentrations between treatments were not significantly different (EXP_stain_: *t-*test=-1.64, df=2, *p*>0.05; EXP_thecas_: *t-*test=-1.94, df=2, *p* >0.05).

**REFERENCES**

1. Frost BW (1972) Effects of size and concentration of food particles on the feeding behavior of the marine planktonic copepod *Calanus pacificus*. Limnol Oceanogr 17: 805–815.
